# A standardized assessment of moldability parameters of thermoplastic materials used in orthotic manufacturing

**DOI:** 10.1371/journal.pone.0267777

**Published:** 2022-08-24

**Authors:** Rodrigo Andrade Martinez, Luciana Bolzan Agnelli Martinez, José Augusto Marcondes Agnelli, Valéria Meirelles Carril Elui

**Affiliations:** 1 Inter Unit Post Graduate Program in Bioengineering (Escola de Engenharia de São Carlos, Faculdade de Medicina de Ribeirão Preto e Instituto de Química de São Carlos), Universidade de São Paulo, São Carlos, São Paulo, Brazil; 2 Department of Occupational Therapy, Universidade Federal de São Carlos, São Carlos, São Paulo, Brazil; 3 Materials Engineering Department, Universidade Federal de São Carlos, São Carlos, São Paulo, Brazil; 4 Department of Health Sciences, Faculdade de Medicina de Ribeirão Preto, Universidade de São Paulo, Ribeirão Preto, São Paulo, Brazil; National Textile University, PAKISTAN

## Abstract

**Objective:**

To establish parameters for standardized assessment of the moldability of thermoplastic materials used in orthotic manufacturing and to develop tests for quantification of moldability parameters by simulating the demands of clinical practice, in order to enable accurate and controlled analysis of material properties.

**Primary outcome measurements:**

Two commercially available materials were submitted to tests for standardized measurement of moldability. Results were correlated with manufacturer information. Moldability assessment was based on two parameters (conformation and fit), expressed as percentages.

**Results:**

Tests, standardized molding procedures and measurements were described. Quantitative data (conformation and fit expressed in percentages) were derived from a pilot study comparing Aquaplast-T™ and Ezeform™. Findings of that study revealed that Aquaplast-T™ is more moldable than Ezeform™ and support technical information provided by the manufacturer.

**Conclusions:**

The assessment method described enabled objective and repeatable measurement of the moldability of materials used in orthotic manufacturing and represent a significant advancement in comparative analysis of materials, with potential positive impacts on therapeutic procedures and clinical decision-making. Tests developed in this study can be used to quantify data provided by manufacturers in order to allow their use by researchers and professionals in rehabilitation.

## Introduction

Orthotic manufacturing is one of the pillars of hand rehabilitation. Several studies suggest orthoses are the most versatile applications for pain alleviation, stabilization and protection of vulnerable upper limb tissues and structures [1]. Well-designed orthotic devices enable the accomplishment of major occupational tasks and participation in relevant activities of daily living, and therefore can make a significant difference in people’s lives [2]. The expertise of therapists in handling certain materials plays a significant role in efficient orthotic manufacturing and should include knowledge of material structural properties and characteristics [2–6].

The structure of a material can be evaluated in different way and refers to material composition and respective impacts on behavior [7]. Material properties can be mechanical or physical. Mechanical properties describe the behavior of a given material under load, whereas physical properties include electrical, optical, thermal and chemical characteristics [8,9]. Thorough understanding of physical and mechanical properties contributes to appropriate material selection, a key factor in routine practice in order to combine material characteristics with the expected function of orthoses [10–15].

Since their advent in the 1970s and 1980s, low-temperature thermoplastics have become the materials of choice for upper limb orthoses. These materials are highly malleable at temperatures ranging from 45°C to 70°C. Their predominance has been acknowledged by several authors and has been credited to features such as service temperature (which facilitates handling and direct application to the skin), conformability (or moldability), adherence (or self-adherence), durability, shape memory and finish (11,12,16–25). Appliances made of low-temperature thermoplastic materials can be tailored to specific patient needs and demands. Therefore these materials are the most commonly used in the manufacture of functional orthoses and other assistive technology devices [1,2,16–20]‬‬‬‬‬‬‬‬‬‬‬‬‬‬‬‬‬‬‬.‬‬‬‬

Moldability reflects the ability of a given material to conform to the anatomy of the region of interest and is one of the most important thermomechanical properties for therapists and researchers in this field [5]. This property enables the design of custom-made orthoses and simplifies the manufacturing process.

The incorporation of low-temperature thermoplastic materials into clinical practice has led to several clinical and laboratory studies designed to evaluate their properties and support their practical application. In the first studies, the moldability, durability and rigidity of different commercially available products were tested in order to determine their behavior and quantify their characteristics [21,22]. Ever since, other studies have been carried out to explore the properties and practical applicability of thermoplastics in orthotic manufacturing in an effort to maximize their use in rehabilitation (18,25,31–33). One such example is mechanical performance, which can be improved through reinforcements with fiberglass, carbon or aramid fibers, cross-linking or polymer blends [23,24]. Low-temperature thermoplastic products are developed from a polymeric matrix, according to the amount of polymers, fillers, reinforcements, resins and elastomers. All of these elements influence material properties and are relevant for routine clinical practice. Polycaprolactone (PCL) and Trans-Poly-Isoprene (TPI) are the two most widely used polymers in orthotic manufacturing, particularly PCL, a biodegradable, biocompatible, non-toxic and easily moldable material [23,25–28]. ‬‬‬‬‬‬‬‬‬‬‬‬‬‬‬‬‬‬‬‬‬‬‬‬

The practical experience of therapists with certain materials is important for orthotic manufacturing [12]. Development of empirical tests based on qualitative approaches have also been reported [29,30]. However, standardized laboratory tests are needed. Guidelines published by the American Society for Testing and Materials (ASTM), the most important North American organization in material testing, are adopted by several countries. Standards set by the International Organization for Standardization (ISO) [4] are also widely used, particularly in Europe [10]. With regard to thermal characterization of materials for orthotic manufacturing, including base polymer (i.e. polycaprolactone) analysis, different techniques have been developed, such as Differential Scanning Calorimetry–DSC [31], Vicat Softening Temperature [32] and Thermogravimetry–TG [23,33–36]. Three-point bending test [37] can be used to determine material rigidity [34,36], whereas tensile testing [38] is thought to be appropriate to assess stretching resistance in clinical settings [39]. Mechanical characterization using compression testing or Dynamic Mechanical Analysis (DMA) [40] has also been described [21,23,36,39].

A fore mentioned standardized tests are not specific for molding capacity assessment. Evaluation of this property is often based on ease of handling and visual inspection of the molded product in clinical practice [29]. In addition to subjective perception, objective assessment is required for deeper understanding and comparative analysis of the molding capacity of existing materials. This study set out to establish parameters for standardized assessment of moldability parameters of thermoplastic materials used in orthotic manufacturing. Simulation tests were designed for accurate determination of material properties in a controlled environment.

## Materials and methods

This is an exploratory study aimed at designing tests to measure the moldability of thermoplastic materials and developing standardized procedures to evaluate this property.

### Determination of technical parameters for moldability assessment

Moldability is a structural property that allows materials to be molded into items of different sizes and geometric dimensions. Moldable materials can be shaped to conform to anatomical regions of interest with even pressure distribution. Molding is often performed at high temperature and/or pressure and can be achieved manually or using shaping machines [4,7,8,41].

Tests designed to evaluate moldability are based on two different parameters: conformability and fit. In conformability testing, a spherical object is used to apply pressure onto a given material in order to simulate the pressure exerted by the therapist during orthotic manufacturing, whereas in fit testing materials are molded onto a cylindrical surface under the effect of gravity.

### Tests development

Standardized tests and procedures were developed to simulate the handling of thermoplastic materials in clinical practice in order to enable reproducible and objective measurement of moldability.

Two test apparatuses were designed using CAD (Computer Aided Design) to allow replication. Three dimensional printing technology (Dimension ELITE 3D Stratasys®) and CatalystEX software were used, as per the Fused Deposition Modeling (FDM) technique.

**Fig 1:**
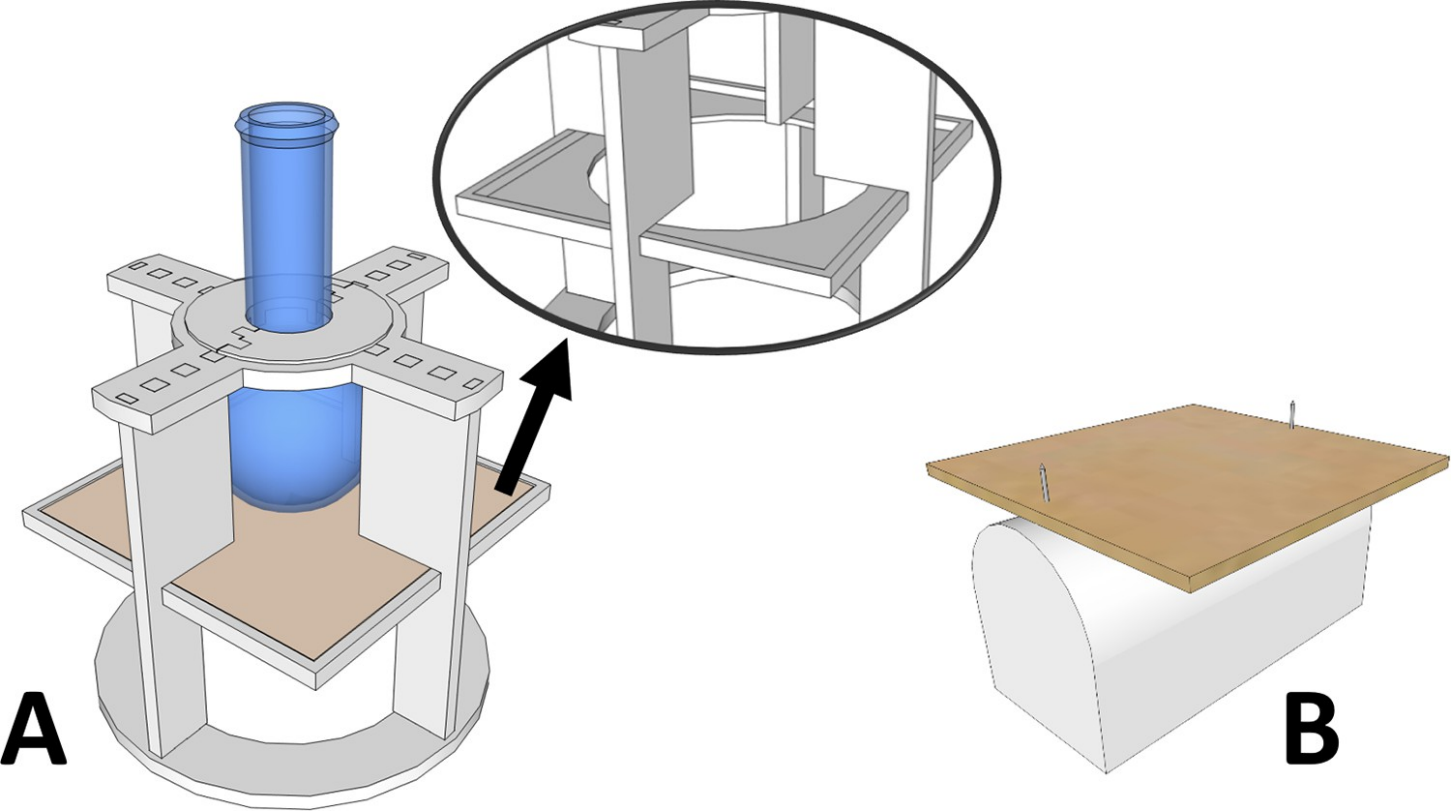
A) Conformability testing apparatus. The test specimen has been removed to expose the hole (shown in detail). B) Fit testing apparatus.

Following apparatus development, trials were carried out to establish testing procedures and criteria (equipment, size of test specimens and measurement variables). A pilot study revealed the need for modifications. Therefore, new models were designed and retested. The final versions of test apparatuses are shown in Fig 1.

### Conformability testing apparatus

The conformability testing apparatus consisted of support containing a flat, square plate designed to hold the test specimen. This plate comprised a central circular hole to accommodate a spherical object (8 cm and 5 cm in diameter respectively) (Fig 1A). The apparatus was designed to maintain the spherical object centered whilst exerting pressure on the test material. Preliminary tests with some commercially available thermoplastic materials revealed that a load of 300 grams would be appropriate to induce deformations within the measuring range of the apparatus. Therefore, this load was selected.

### Fit testing apparatus

The fit testing apparatus consisted of half cylinder support (5 cm in diameter and 10 cm in length) containing one nail on each side to hold the test specimen in the desired position (Fig 1B).

### Pilot study

The final version of each apparatus was used to test two low-temperature thermoplastic materials available in the market and widely used in orthotic manufacturing: Rolyan® Ezeform™ and Rolyan® Aquaplast-T™. Materials from the same manufacturer were used to facilitate comparisons. Availability in the Brazilian market was as also accounted for in material selection. Each test was repeated five times with different test specimens of the same material. Mean values and standard deviations were calculated and used in the analysis.

Pilot studies were carried out at the Polymer Laboratory of the Universidade Federal de São Carlos, in climatized room (23°C).

The following materials and equipments were used:

- Apparatuses developed for conformability and fit testing;
- Five test specimens (10 cm x 10 cm) of each material;
- Thermostatic water tank (TermoULTRA 3000W, H2heater)
- Digital caliper with 0.01 mm accuracy (Mitutoyo Absolute Digimatic 150 mm);
- Infrared thermometer (Benetech, model GM900);
- Timer.

Apparatuses were assembled and test specimens attached. This step was followed by immersion in water heated to the service temperature of test materials and molding. Measurements were made after removal from the water and cooling to room temperature.

## Results

This is an innovative, exploratory study involving two tests designed for standardized assessment of the moldability of low-temperature thermoplastic materials. Tests developed in this study are a step forward towards standardized, objective measurement of moldability assessment parameters. Methods developed and data derived from pilot tests are also important contributions.

### Conformability testing

**Fig 2:**
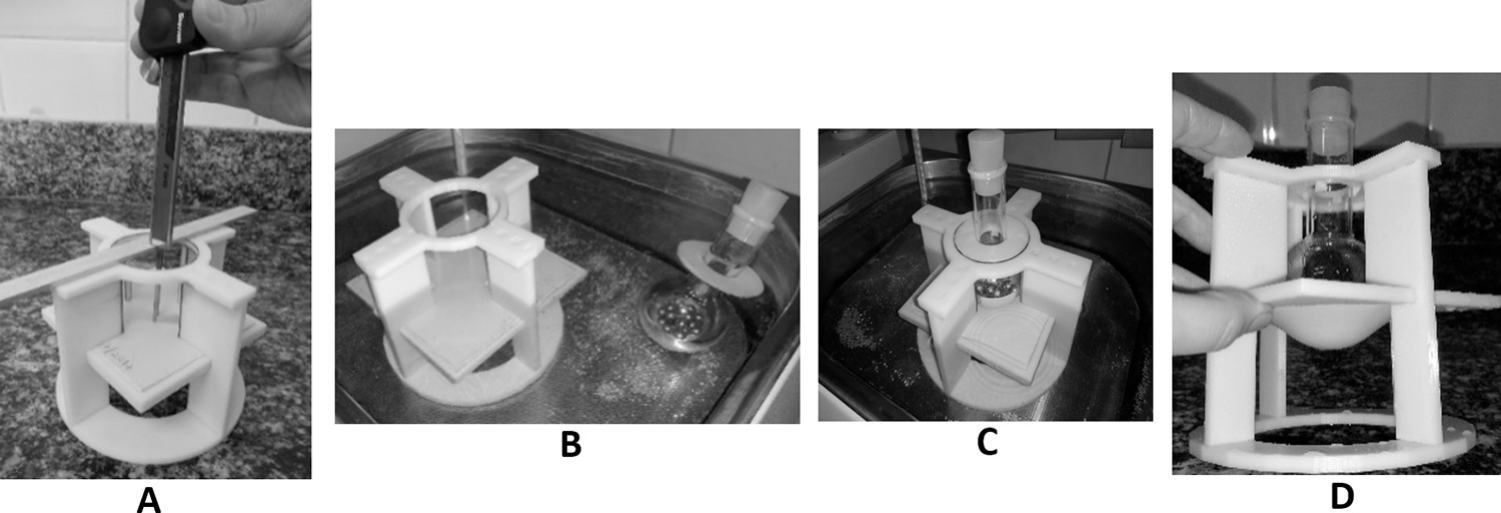
Conformability testing: A) Measurement made with the aid of a metal rod; B) Heating of the assembly and spherical object in water bath; C) Spherical object resting on top of the test specimen; D) Removal from the water bath and cooling.

In order to accommodate and eliminate initial deformations, the test specimens was mounted onto the apparatus, immersed in water heated for 5 minutes. Following removal of the assembly from the water and cooling to room temperature, baseline measurements (i.e., unloaded) were made at the central portion of the test specimens, where deformation is more intense. A metal rod was used to support the caliper (Fig 2A). Next, the assembly and the 300-gram spherical object were heated separately by immersion in water for 5 minutes (Fig 2B). While still in the thermostatic bath, the spherical object was placed in the center of the hole, where it remained in contact with the test specimen (Fig 2C) for another 5 minutes. In order to allow additional deformation without the effect of buoyancy, care was taken not to dislodge the spherical object during removal of the assembly from the water bath (Fig 2D). The molded test specimen was allowed to cool to room temperature (23°C). Temperature was confirmed using an infrared thermometer. Finally, the spherical object was removed and measurements made, as shown in Fig 2A.

Differences between baseline and final measurements of deformation were calculated. The larger the difference, the more moldable the material. Moldability can be expressed as a relative value. Conformability test results were interpreted as follows: 0%, no difference between baseline and final measurements; 100%, 50 mm difference between baseline and final measurements (i.e., diameter of the spherical object).

### Fit testing

**Fig 3:**
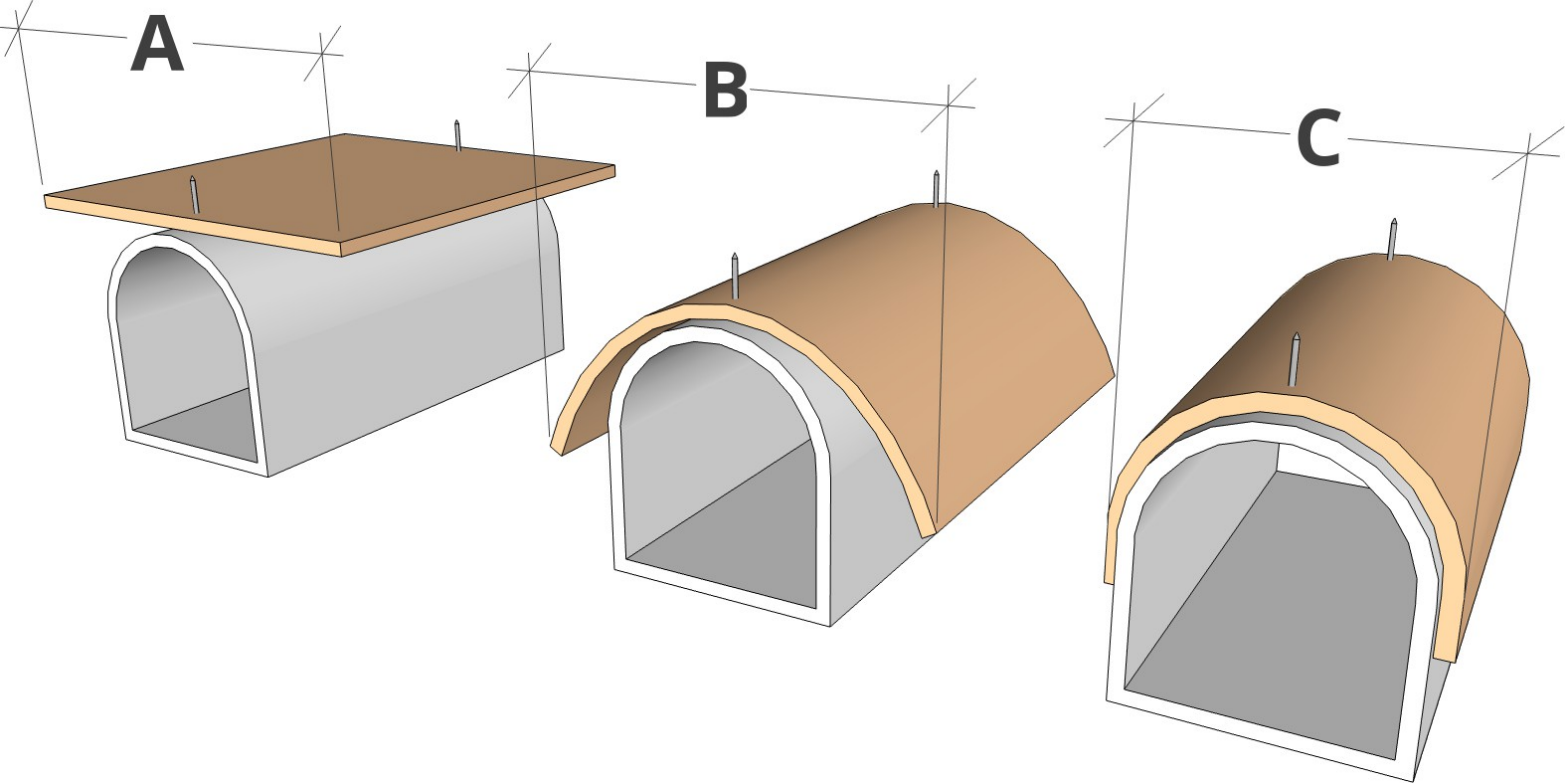
Measurement method used in fit testing. A) no fit (0%); B) intermediate fit; C) full fit (100%).

Using the nails on each side, the test specimen was firmly attached to the curved surface of the support. A digital caliper was used to measure test specimen width prior to and after testing. Measurements were made in the direction of test specimen deformation. Test execution steps and the measurement method are shown in Fig 3.

**Fig 4:**
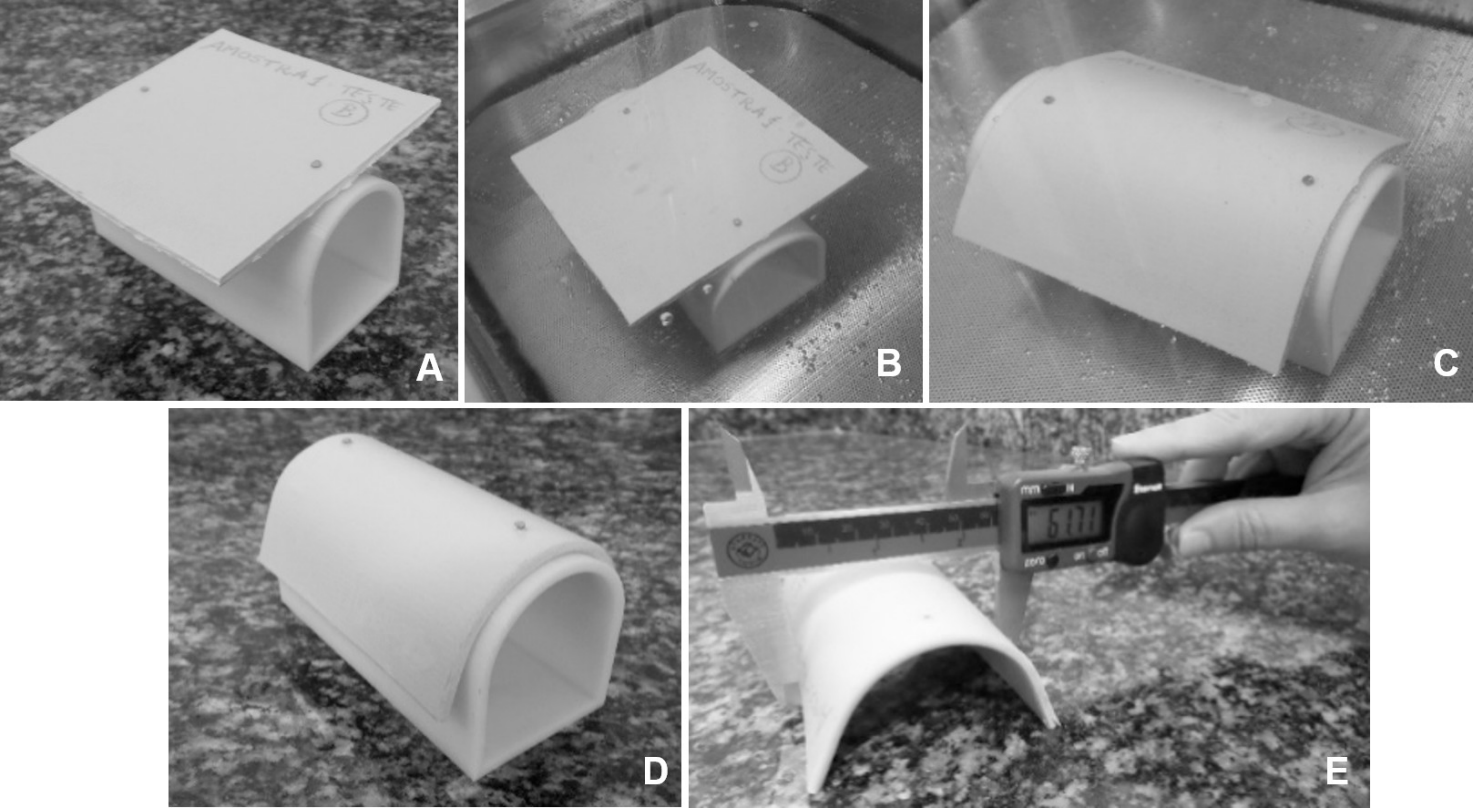
Fit testing apparatus: A) Attachment of the test specimen B) Immersion of the assembly in heated water; C) Assembly immersed in water bath for a full five minutes; D) Removal of the assembly for cooling; E) Width measurement.

Prior to testing, the apparatus was assembled and the test specimen attached. Care was taken to ensure contact between the top portion of the support and the midline of the test specimen (Fig 4A). This was followed by immersion in water heated to the service temperature of the test material for 5 minutes (Fig 4B and 4C). The assembly was carefully removed from the water and allowed to cool to room temperature (23°C) (Fig 4D). The test specimen was then detached and the width measured. Measurements were made on a flat surface (table or workbench) using a digital caliper (Fig 4E).

### Pilot study findings

Application of tests developed to two low-temperature thermoplastic materials (Rolyan trademark) according to standardized procedures enabled the quantification of both moldability parameters described in this study: conformability (under a load of 300 grams) and fit (unloaded). Conformability and fit (%) calculated for each product tested in this study were consistent with information provided by the manufacturer (Table 1).

**Table 1:** Comparisons between manufacturer information and data derived from standardized assessment of moldability.

	Aquaplast-T™	Ezeform™
**Conformability** **(300 g load)**	55% ± 1%	34% ± 1%
**Fit** **(5 cm diameter cylinder)**	100% ± 0%	77% ± 6%
**Manufacturer Information**	Offers the optimum combination of intimate conformability and resistant stretch. [42]	Extremely strong and durable, tolerates handling well. Maximum resistance to stretch with superior draping and conforming qualities. Stays in place while critical contours are moulded. [43]

Findings of this study support the applicability of tests designed for quantitative analysis of moldability.

Pilot test results (including sample standard deviation) can be used to determine sample size (n) in future studies, given the desired confidence level and acceptable difference.

## Discussion

**Fig 5:**
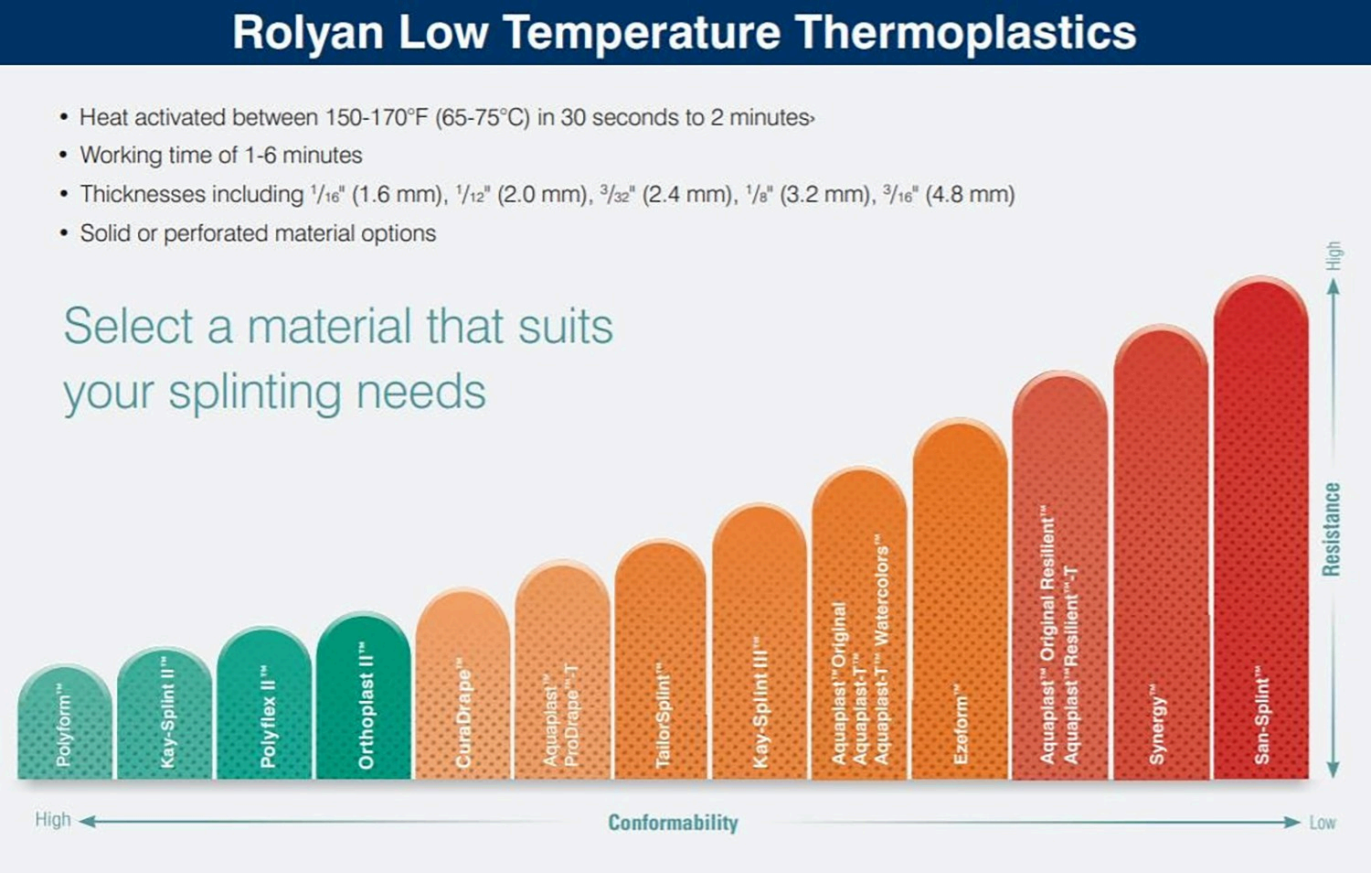
Scale of conformability and stretching strength of Rolyan® products [44].

A wide array of low-temperature thermoplastic materials for orthotic manufacturing can be found in the market. In this study, tests were carried out with two different commercially available materials. Products made by the same manufacturer were selected in order to facilitate comparative analysis. In order to validade the reliability of tests, manufacturer information regarding handling characteristics was reviewed. Conformability and stretching strength data available in Rolyan® product online catalogues (Fig 5) indicate Aquaplast-T™ is a more moldable material than Ezeform™.

Manufacturer information (Fig 5) does not include numerical data. However, materials are presented in increasing order of conformability and, therefore, can be compared and correlated with findings of this study. Conformity and fit test results (Table 1) revealed that Aquaplast-T™ has higher moldability than Ezeform™, as shown in the scale provided by the manufacturer.

The value of tests developed in this study lies in the fact that they enable objective measurement of moldability, one of the most important properties for application of thermoplastic materials in orthotic manufacturing. Moldability reflects the plasticity of a material. Therefore, plastic materials are expected to be easily moldable and to conform to bony prominences, especially in the wrist and hand. Moldability is crucial to provide comfort and can be a determining factor in custom-made orthoses [2].

Strategies to assess the moldability of thermoplastics for orthotic fabrication have been provided by manufacturers and can be found in the literature. However, these consist primarily of descriptive information and online catalogues and often display generic data, including use indications. In some cases, materials are categorized into subgroups or scales to facilitate distinction between them [17,18,45]. Sadly, scoring systems aimed at evaluating the moldability of different products are lacking. Such scores could be a valuable tool to assist therapists in material selection. Materials such as Aquaplast-T™ and some Orfit® thermoplastics become transparent when heated. In these cases, the contact surface, including bony prominences and susceptible pressure points, can be seen early in the molding procedure and moldability more easily estimated [18,45].

Tests proposed in this study enabled the quantification of conformability and fit and revealed that Aquaplast-T™ has higher conformability and better fit than Ezeform™. This does not mean one material is superior to the other. Rather, it indicates a difference in molding characteristics which must be accounted for in different circumstances.

Moldable materials provide excellent reproduction of anatomical details and remain in the desired shape with no need to apply pressure during cooling [1,17]. On the flip side, highly malleable materials can be difficult to handle in fabrication of larger orthoses for extensive areas of the body. In these cases, more rigid materials may offer better conformability with minimum stretching [45]. Appropriate understanding of the mechanical properties of products available in the market facilitates material selection and contributes to efficient handling and application, leading to optimal outcomes [12].

Given the complexity of human anatomy and the wide range of orthopedic devices, moldability requirements for clinical application are thought be greater than for tests like the ones developed in this study. These tests represent a first step towards a standardized, objective and repeatable measurement method for comparative analysis of different products. Further studies with different parameters are warranted to refine the protocol described. One possibility would be to develop an apparatus aimed at quantifying the conformability of different materials to curved and irregular surfaces in different planes in order to complement the assessment of moldability by contemplating a third parameter, which is related to the material property defined as drapability.

Regardless of suggestions regarding the inclusion of additional moldability parameters in the protocol described, results derived from tests designed in this study may help expand the understanding of existing materials and assist professionals in appropriate product selection. Quantification of material properties, as in this work, represents an advancement in the classification of and differentiation between existing materials. Also, the fact that tests developed do not required direct handling of materials by therapists/researchers ensures unbiased evaluation and faithful replication, with appropriate standardization throughout the testing procedure, from molding to final measurements (i.e., after cooling). According to Garcia, Spim & Santos (2000) [46] and Canevarolo (2019) [8], material testing contributes new information to support the development and/or modification of manufacturing processes.

In the case of relatively complex orthoses, objective tests can be combined with subjective evaluation by therapists or researchers, whose practical experience should be acknowledged and taken into account. Standardized measurements are critical to establish a common language between professionals from different fields [4], and can provide data to support anecdotal evidence. This line of thought is adopted by several authors, who believe the physical properties of polymers can be evaluated using empirical approaches and technical information [8,34].

The relevance of comparative analysis of materials properties (moldability and others) must be emphasized. In a study investigating new thermoplastics for orthotic manufacturing, Meng & Hu (2009) [24] reported that polymer compounds with shape memory and their mixtures may exhibit new properties, which may be significantly different from those of pure polymers. Several studies along those lines of research can be designed to investigate other relevant material properties, such as shape memory, self-adherence and rigidity.

### Study limitations

The small number of materials submitted to standardized testing is a limitation of this study. Further studies are warranted to validate the systematic testing of different commercially available products, including products from different manufacturers. Also, although two parameters (conformability and fit) were evaluated, the contact surface between test material and object and the ability of materials to conform to irregular surfaces were not investigated in this study. Inclusion of a third parameter (research question) and design of a dedicated testing system is warranted to contemplate what is referred to as drape or drapability. The effects of elasticity and memory are other important components worthy of investigation.

## Conclusions

The assessment method proposed in this study enables moldability quantification according to material conformability and fit. Tests are reproducible and represent a significant advancement in comparative analysis of materials, with potential positive impacts on therapeutic procedures and clinical decision-making.

Tests described can be used to assist clinicians and researchers with a particular interest in this type of materials or professionals who use low-temperature thermoplastic materials to fabricate orthoses. These tests may also provide quantitative data for manufacturer information about material properties (conformability and fit), which should be systematically validated in orthotic manufacturing.

This study was made possible by the collaboration between occupational therapy and engineering researchers. Interdisciplinary collaboration plays an important role in innovative research and development of healthcare resources and technology.

This study is scientifically relevant and provides tools for in-depth, standardized, reproducible analysis of some of the most important properties of thermoplastic materials, with significant contributions to the expansion of knowledge in the fields of rehabilitation and engineering.
